# Evaluation of a Bioabsorbable Scaffold and Interlocked Nail System for Segmental Bone Defect

**DOI:** 10.3390/jfb14040183

**Published:** 2023-03-27

**Authors:** Morshed Khandaker, Reuben Lane, Shannon Yeakley, Hussein Alizereej, Sadegh Nikfarjam, Abdellah Ait Moussa, Melville B. Vaughan, Amgad M. Haleem

**Affiliations:** 1 School of Engineering, University of Central Oklahoma, Edmond, OK 73034, USA; 2 Department of Biology, University of Central Oklahoma, Edmond, OK 73034, USA; 3 Department of Orthopedics, University of Oklahoma Health Science Center, Oklahoma City, OK 73104, USA

**Keywords:** bone, interlock nail, polycaprolactone, scaffold, segmental bone defect

## Abstract

In the current study, we designed and manufactured a scaffold and fixation system for the reconstruction of long-bone segmental defects in a rabbit tibia model. We used biocompatible and biodegradable materials, polycaprolactone (PCL) and PCL soaked with sodium alginate (PCL-Alg) to manufacture the scaffold, interlocking nail and screws using a phase separation casing method. Degradation and mechanical tests on the PCL and PCL-Alg scaffolds indicated that both were suitable for faster degradation and early weight-bearing capacity. PCL scaffold surface porosity facilitated the infiltration of alginate hydrogel through the scaffold. Cell viability results showed that the number of cells increased on Day 7 and decreased marginally by Day 14. For accurate placement of the scaffold and fixation system, a surgical jig was designed and 3D-printed using biocompatible resin in a stereolithography (SLA) 3D printer, then cured with UV light for increased strength. Our cadaver tests using New Zealand White rabbit confirmed our novel jigs’ potential for accurate placement of the bone scaffold, intramedullary nail and the alignment of the fixation screws in future reconstructive surgeries on rabbit long-bone segmental defects. Additionally, the cadaver tests confirmed that our designed nails and screws were strong enough to carry the surgical insertion force. Therefore, our designed prototype has the potential for further clinical translational study using the rabbit tibia model.

## 1. Introduction

Long-Bone segmental defects are bone voids that stay unfilled without surgical intervention [1]. They can be the result of trauma, tumor or infection [2]. The reconstruction of segmental bone defects is a major challenge in orthopedic surgery owing to the biological limitations associated with autologous bone grafts [3]. In the current study, we aim to mitigate this shortcoming with a 3D-printed scaffold and fixation system that will supplement cell growth and secure the placement of the scaffold over the defect area. The scaffold fixation consists of a combination of an intramedullary nail and transfixion screws. This type of fixation has proven successful in several in vivo animal long-bone reconstruction surgeries [1,3], and in the current study, it was tested on a rabbit tibia, which is a relatively much smaller long-bone than those previously repaired by this technique. The scaffold and interlocking nail system implant are intended to fill the void left by the bone defect and stimulate tissue growth across a critical-size long bone defect while providing mechanical support for weight-bearing activities.

Current surgical approaches to treating long-bone segmental defects are extremely invasive, require multiple surgeries and a long recovery time, and most often do not allow for load bearing [4]. Some of the currently employed treatment options include distraction osteogenesis, induced membrane and vascularized fibular transplantation [5]. They each have advantages, drawbacks and complications. Another treatment option uses halo-type equipment to slowly shorten the limb so that the ends of the segmental bone defect become close enough for the body to repair [6]. Unfortunately, this process has been shown to result in a permanently shorter limb. Current treatment options do not use scaffold implants; however, numerous studies are looking into their use with various animal models.

Scaffolds are materials designed to contribute to the development of new functioning tissues for medicinal reasons by causing desired cellular interactions [6]. Seeding cartilage cells onto bone spicules by Green in early 1970 was the first attempt at tissue scaffolding [7]. Since then, seeding cells on properly engineered scaffolds from biocompatible biomaterials have been proposed for new tissue formation [8]. Interlocking nail systems have been developed and tested for large animals, such as cows, sheep and dogs, but very few of these systems currently exist for use with animals as small as a rabbit, and only a handful focus specifically on the rabbit tibia [9,10]. These existing systems use interlock nails and locking screws composed of stainless steel or titanium alloy [9,11].

Osteoblast, endothelial and stem cells are frequently seeded onto 3D-printed photopolymerizable hydrogel structures such as Poly (ethylene glycol) (PEG)-based hydrogels that are capable of supporting the creation of three-dimensional tissue [12]. Cell attachment to the 3D-printed structure was not good due to the gaps between scaffold layers being too large for cells to fill, so they sank to the bottom and attached to the plate. In this respect, various research studies have tested cell growth in 3D-printed sodium alginate hydrogel scaffolds [13]. Grandi et al. [14] used 3D-printed scaffolds that had been treated or covered with a hydrogel. The cell viability of MC3T3-E1 murine preosteoblastic cells on PCL-alginate was better than those without hydrogel treatment (PCL only). This is because hydrogel filled the gaps in the 3D-printed layered structures and acted as the extracellular matrix, which is necessary for cell attachment. Furthermore, hydrogel provides a more convenient environment for the growth of cells due to the nutrient supply from which the hydrogel is composed. For the above reason, this study used a porous PCL-alginate structure to develop a scaffold for the repair of a segmental bone defect.

Polycaprolactone (PCL) is widely used for applications in tissue engineering and drug delivery [14,15]. Three-dimensional (3D) modeling of long bone scaffold and interlock nail of rabbit can be made using micro-computed tomography [16]. PCL-based scaffold has been used as bone substitute for bone fracture fixation [17]. To our knowledge, none of the available research studies have utilized a PCL-based scaffold and fixation system for healing segmental bone defects on a rabbit tibia. Along the same lines, current methods for implant nail fixation are neither standardized nor consistent across studies. The need for biocompatible targeting jigs to accurately implant the nail within the medullary cavity and to track the correct placement of screws through the nail is necessary during surgery. Unfortunately, jigs for animals smaller than cats are currently not available on the market and a custom-targeting jig for delivering scaffolds, nails and screws should be designed and manufactured.

As of now, no standardized treatment methods exist for critical-size long-bone segmental defects in humans. Current solutions remain unsatisfactory, requiring multiple complex surgeries, with a high risk of infection, and often leaving patients with lifelong difficulties. A new solution that promotes bone union, allows weight bearing during the healing process, and requires only minimally invasive surgeries should be developed and tested. The current study is the first step toward potential future human applications. The aspects of the proposed solution that make it unique include the reduced size of the intramedullary nail and fixation screws and the bioabsorbable nature of the scaffold and fixation system. The combination of an intramedullary nail and scaffold fixation will permit load bearing soon after surgery and at the same time will eliminate the need for follow-up surgeries that otherwise would be required if non-bioabsorbable implants were used. It will also allow bone cells to gradually fill the space left by the implants as they dissolve over time.

This study augmented PCL-based scaffolds for producing osteoconductive bone substitutes combined with a highly biodegradable hydrogel for critical-size bone defects. The study results will provide proof of the principle and pre-clinical efficacy of the biological activity and cytocompatibility of PCL-based bone substitutes in vitro. Additionally, the effectiveness of bone substitute and nail implanting mechanisms on a rabbit tibia model through a unique targeting jig was tested through cadaveric study.

## 2. Materials and Methods

### 2.1. Materials

The scaffold was made from polycaprolactone (PCL) (pellet size ~3 mm, average Mn 80,000), which is a USA federal drug administration-approved biomaterial. The nails and screws were also cast using PCL. The final products of this study included the scaffold implant, the interlock nail and screws (interlocking nail system) and a targeting jig. This study used PCL pellets and tetrahydrofuran (THF) solution for making the PCL-based scaffold, interlock nails and screw. Sodium alginate, calcium chloride and phosphate buffered saline (PBS) were used to prepare sodium alginate hydrogel. All chemicals were purchased from Sigma-Aldrich (Sigma-Aldrich Co., LLC., St. Louis, MO, USA).

### 2.2. Sample Design

**Figure 1:**
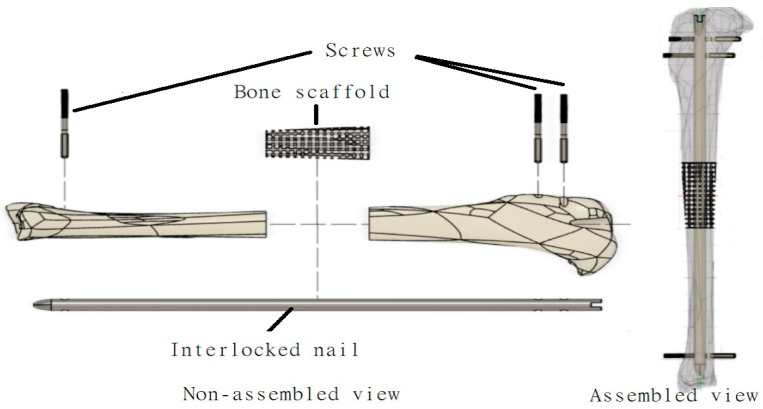
Schematic representation of various components (bone scaffold, interlock nail, screw) designed in this study to repair a long bone defect using a tibia model.

This study designed samples for in vitro and ex vivo studies. For in vitro studies, 10 mm diameter and 5 mm height PCL and PCL-Ag samples were prepared to determine the morphological, mechanical and biocompatibility of each of the samples. All samples were created using phase separation casting methods with polycaprolactone (PCL) as the scaffolding material and sodium alginate hydrogel as the coating material of the scaffold. For ex vivo studies, this study designed a bone scaffold, an interlocking nail and screw to repair a critical size bone defect of a rabbit tibia (Figure 1). PCL-based scaffolds, nails and screws were only used for the efficacy of targeting jigs by 3D-printed rabbit knees and rabbit cadavers, as those studies did not involve cells.

#### 2.2.1. 3D-Modeling of Bone Scaffold, Interlock Nail and Screws

**Figure 2:**
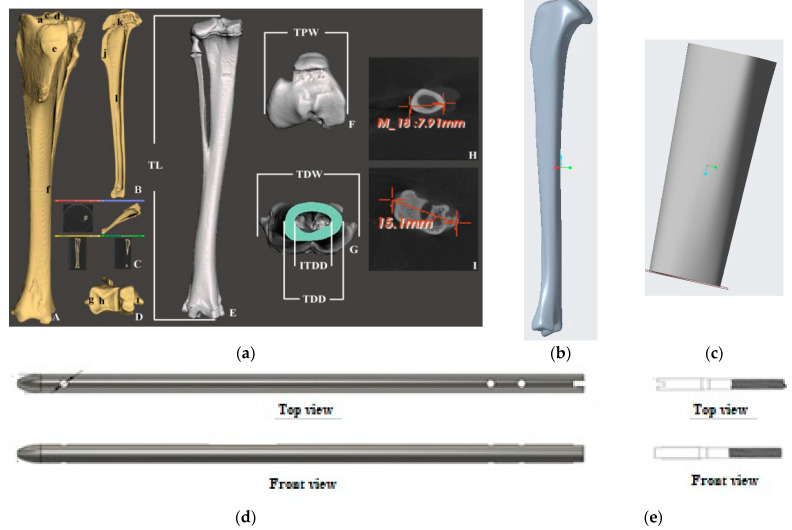
(**a**) 3D prototyping of bone scaffold, interlock nails and screw of a rabbit tibia model based on dimensions given in Bakici et al. [16]. Our designed solid model of (**b**) rabbit tibia, (**c**) bone scaffold, (**d**) interlock nail and (**e**) screw.

The development of the interlocking nail system for the rabbit tibia had three major design considerations: weight-bearing ability, biodegradability and ease of implantation. These factors influenced the design of the interlock nail and screws, material selection and choice of production method. Dimensions for the interlock nail and interlocking screws were initially based on the average measurements of rabbit tibias. Particularly, a study by Bakici et al. [16] (Figure 2a) found that the average tibial length (TL) of male and female New Zealand rabbits ranged from approximately 100.02 mm–107.78 mm, while the internal tibial diaphysis diameter (ITDD) (indicating the diameter of the medullary cavity) ranged from 4.17 mm–4.39 mm, and the tibial proximal width (TDW) (indicating maximum distance across the proximal surface) ranged from 16.61 mm–18.16 mm. Following these results and anticipating the need to fit within the medullary cavity, the interlock nail was designed to be 3.5 mm in diameter with a length of 110 mm. A “bullet nose” geometry for the distal end of the nail was utilized to aid implantation into the medullary cavity, while the proximal end was designed with a hole to be attached to the targeting jig. Two 1.5 mm centrally smooth screws were designed for locking the proximal end, while only one screw was used for the distal end to avoid a fracture in thinner sections of the tibia [9]. We used Solid Work software to model the rabbit tibia based on the above dimensions (Figure 2b). A 20 mm bone was cut from the model using the software to make the 3D model of the bone scaffold (Figure 2c).

#### 2.2.2. Targeting Jig for Delivery of Interlock Nail System

**Figure 3:**
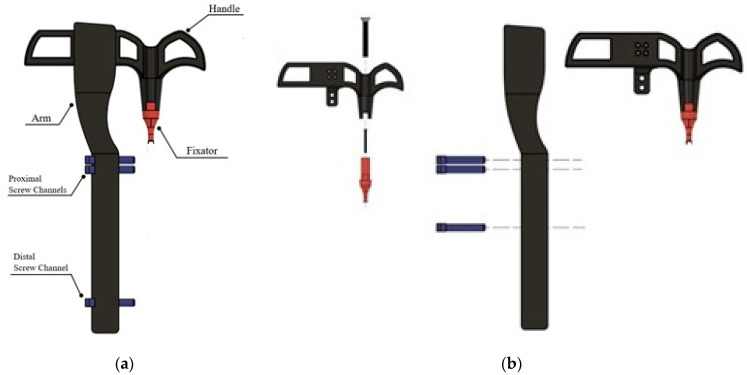
Designed targeting jig for delivering various components to repair long bone defects (bone scaffold, interlock nail, screw) designed: (**a**) assembled and (**b**) exploded views.

A rabbit tibia model was also developed and printed to test and demonstrate implantation of the interlocking nail system and scaffold. An open-source CAD file of a rabbit tibia model was used as the base [18]. Channels for the nail and screws, as well as a 20 mm segmental defect, were pre-modeled before printing using Formlab SLA BioMed Amber Resin. A custom targeting jig was designed for minimally invasive implantation of the interlocking nail system with little to no visual assistance during surgery. The design included a fixator piece locked to the end of the nail at a specific angle using a 2 mm screw, a handle attached to the fixator for manually inserting the nail into the medullary cavity, and an alignment arm with proximal and distal screw channels to ensure proper placement of screws into the pre-drilled holes of the nail. The jig parts were modeled and assembled using Solid Works 3D CAD design software, as shown in Figure 3.

### 2.3. Sample Preparation

For the purposes of this project, a biodegradable material with appropriate strength for weight bearing and adequate compressive modulus to avoid stress shielding was desired. The material chosen to cast the scaffold, nail and screws was PCL as used by Wong et al. [17] due to its biodegradability and similar mechanical properties to bone. This casting process followed the exact steps of Wong et al. [17]. The PCL bone scaffold was coated with alginate to prepare PCL-Alg with the goal of increasing cell attachment and viability. Next is the preparation methods for PCL and PCL-Alg structures.

#### 2.3.1. PCL

**Figure 4:**
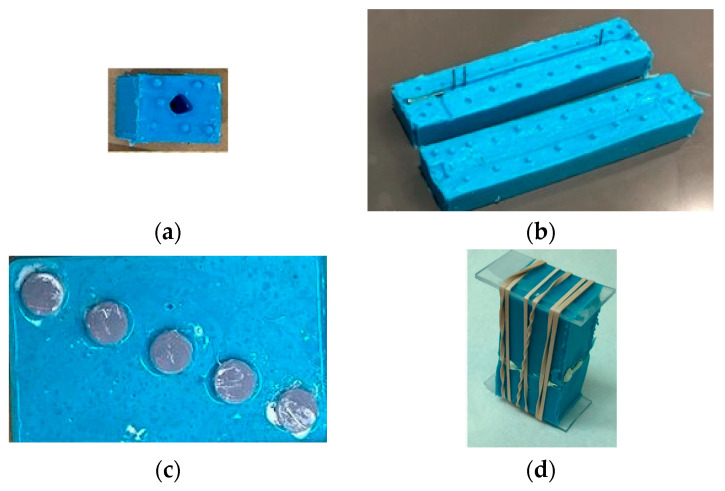
Silicone molds used for making (**a)** bone scaffold, (**b**) interlock nail and screws, and (**c**) in vitro test samples for interlocking nails with bone. (**d**) The two-part silicone molds during the curing of PCL gel.

This study used the Phase Separation Casting Method [14] to prepare PCL scaffolds. We used a 10-mm diameter and 5-mm height silicone mold available in the lab to prepare all in vitro samples. For ex vivo samples, a 20-mm scaffold was modeled from a rabbit tibia model and resin (SLA) printed, cured with UV light for 2 h to remove excess sulfur, and brushed with a lubricant. Two-part silicone molds with embedded rods (to ensure a hollow center) were created using the resin scaffold as a master, as shown in Figure 4a. Resin-printed master pieces made of photopolymer resin were chosen to create negative molds due to the high level of precision observed with this material and printing method. Silicone was used to create a two-part mold for casting the scaffolds (Figure 4a) and nail and screws (Figure 4b) with the help of rubber bands. Additional holes were added to the molds to accommodate rods for securing the molds in place during curing. Figure 4c shows the silicone mold used for making the in vitro test samples. Figure 4d shows the fastened molds for casting a bone scaffold.

**Figure 5:**
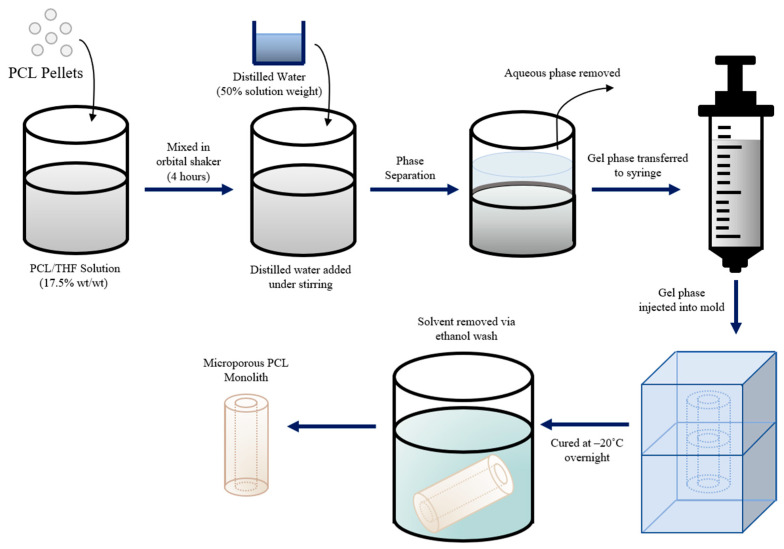
Schematic representation of the phase separation casting technique used to produce PCL scaffolds.

Casting utilized a phase separation technique detailed in a study by Grandi et al. [14] and Coombes et al. [15], as illustrated in Figure 5. In brief, PCL pellets were dissolved in 10 mL of tetrahydrofuran (THF) at a ratio of 17.5% wt/wt (weight of PCL per weight of THF) by constant agitation in an orbital shaker until a uniform solution was obtained. A volume of distilled water equivalent to 50% of the solution’s weight was added while stirring until two phases formed: an aqueous phase and a gel phase. The aqueous phase was discarded while the gel phase was transferred to a syringe and injected into a silicone mold (pre-cooled to −20 °C) and left to cure at −20 °C overnight. After curing, the scaffold was removed from the mold, washed in ethanol to remove the solvent, and allowed to dry at room temperature, yielding a microporous PCL scaffold.

#### 2.3.2. PCL-Sodium Alginate

The sodium alginate (Alg) hydrogel solution was created for coating a PCL scaffold with alginate using a ratio of 1 g of sodium alginate powder to 40 mL of phosphate-buffered saline (PBS). These materials were put in a beaker and placed on a magnetic stirrer (Corning PC-620D, Corning Inc., Corning, New York, NY, USA). A magnetic stir bar was put in the beaker and the settings of the hot plate stirrer were set to 150 rpm and 90 °C for 2 h. Once this gel was made, the beaker was covered in parafilm and placed in the incubator until it was ready for use. To prepare the PCL-Alg scaffolds, the scaffolds were soaked in gel for 5 min and then submerged in a calcium chloride solution. This step is necessary because it allows the calcium ions and alginate polymers to cross-link, which causes the gel to become a semi-solid gel [13].

### 2.4. Experiments

#### 2.4.1. Morphological Examination

To observe the bone scaffold surface morphology, the top surfaces of PCL and PCL-Alg scaffolds at arbitrary locations were viewed using a Hitachi TM 3000 SEM (Hitachi High-Technologies Corporation, Nake, Japan). The views were used to compare the scaffolds qualitatively. We used the MATLAB image-processing algorithm to find the total pore area. We used USA National Institute of Health ImageJ software (Version 1.53) to measure pore size.

#### 2.4.2. Mechanical Tests

**Figure 6:**
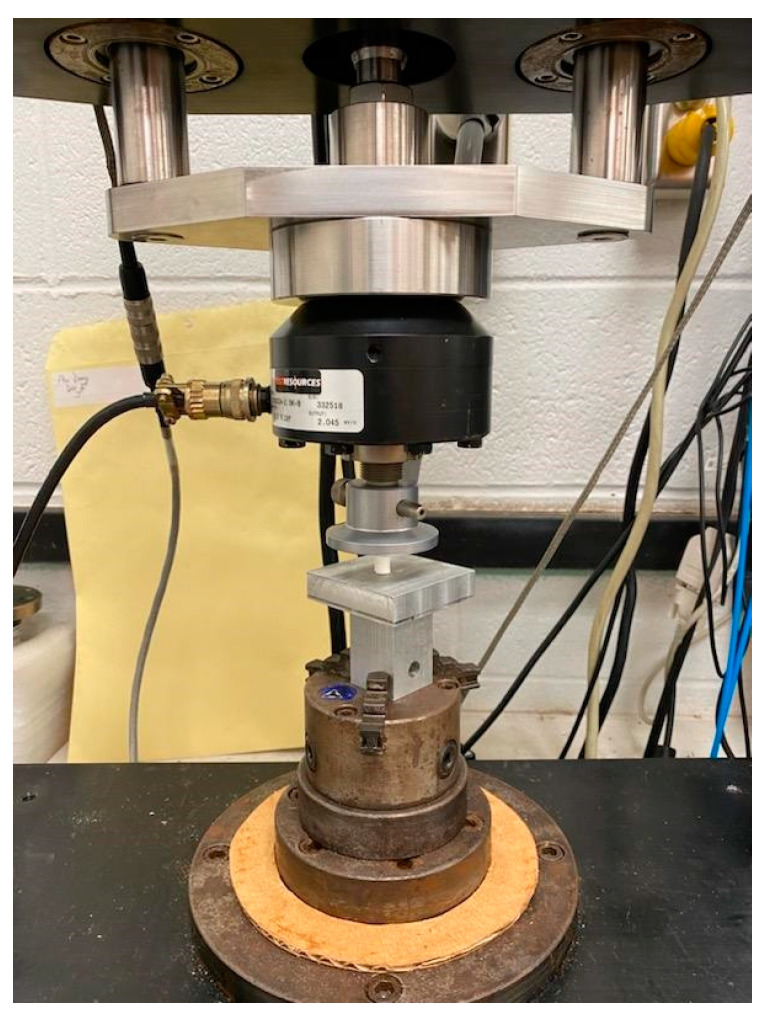
Compression test of a round PCL sample (diameter 9.8 mm and 7.8 mm height) in a Test Resource Universal Testing Machine.

A Test Resources Inc tensile/compression mechanical test system (Figure 6) was used for the compression test of both scaffolds. A silicone mold of 10 mm diameter and 8 mm height was used to cast the scaffold according to the method described in Section 2.3. Each group of samples was mounted between the holders in the test system. The sample was compressed to the scaffold height at a rate of 0.05 mm/sec during the unconfined compression tests. The scaffold load and the corresponding displacement were directly recorded from the testing machine software and exported to Microsoft Excel. The stress (load/cross-sectional area) and strain (displacement/initial height) curves were determined from the load and displacement values. The slopes of the curves were utilized to compare the differences in compressive strength and modulus between the samples. The elastic modulus and maximum stress were reported to evaluate the compression performance for each scaffold. The maximum stress was calculated from the stress–strain curve, defined as the maximum stress point. The elastic modulus was calculated from the slope of the stress–strain curve using the data points until the initial change of sudden displacement. We took the average of the three samples as the test results.

#### 2.4.3. Bioactivity

a. Water Absorption and Degradation Test

The water absorption of PCL and PCL-Alg was assessed by soaking each group of scaffolds in deionized water for one hour. The initial weight (W_0_) of the three samples from each scaffold group was measured. After an hour, the weight of the scaffolds (W_t_) was measured. The value of the PBS absorption rate in percentage was measured using the formula: (W_t_ − W_0_) × 100%/W_0_. A degradation test was conducted using another 6 samples (*n* = 3/group). The samples were submerged in PBS to determine the change in their mass in percentage after 7 days.

b. Cell Cultures

A silicone mold with holes of 10.3 mm in diameter and 5 mm in height, the same as the well dimension of a 48-cell culture well plate (Figure 4c), was used to cast the scaffold for cell culture samples. This study used the mouse osteoblast 7F2 cell line (ATCC CRL-12557) for the cell viability assays. The scaffolds were sterilized by soaking them in 100% ethanol and then drying under UV for 30 min. Three sterilized PCL and PCL-Alg samples, the same diameter as the well diameter, were placed in separate wells in 48-well cell culture plates, and each sample was seeded with 50,000 cells per mL of cell culture medium (25,000 cells per well). The cells were cultured in Dulbecco’s Modified Eagle Medium (DMEM)-high glucose supplemented with 10% fetal bovine serum (FBS) and penicillin/streptomycin/amphotericin. Osteoblast cultures were maintained at 37 °C and 5% CO_2_. Cells harvested in log-phase growth at 80% confluency were used for all the cell culture experiments in this study. The cells with media were seeded on top of the scaffolds. The plates were incubated at 1, 7 and 14 days for cell viability assay.

c. Cell Viability Assay

The biocompatibility of the PCL and PCL-Alg materials was determined using the alamarBlue™ Cell Viability Reagent (Thermo Fisher Scientific, Waltham, MA, USA) to estimate the number of viable cells after various amounts of time (Days 1, 7 and 14) following the protocol recommended by the manufacturer. Briefly, 100 μL of the reagent was added to the scaffold-seeded cell culture media and incubated for 8 h. The alamar blue dye reacts with viable cells in the sample well, which results in a change of color. The change in color and the number of viable cells are proportional. The reading of the colorimetric value was recorded in terms of percent resazurin reduction. A standard curve was found before performing the experiments to be able to calculate the cell number from the percent reduction values and determine the effective cell population range to derive linear correlations. The percent reduction values were determined using the BioTek Synergy H1 Microplate Reader (Agilent Technologies, Inc., Santa Clara, CA, USA) at 570 nm.

### 2.5. Targeting Jig Efficacy Tests

The efficacy of our designed targeting jig was tested using a 3D-printed rabbit tibia model and rabbit cadaveric tests. The rabbit tibia was modeled based on the dimensions provided by Bakici et al. [16]. A six-week-old euthanized female New Zealand White Rabbit (NWR) stored at the University of Oklahoma Health Science Center (OUHSC) animal care facility was used for this study. As per Huang et al. [18], an orthopedic oscillating bone saw created a 20 mm defect (same as scaffold length) in the rabbit tibiae inferior to where the fibula fuses with the tibia. This defect length represents 20% of the tibia’s length and has been used by others as a measure of a critical-sized defect [19]. Prior to insertion in the tibia, a series of drill bits (1 mm, 2 mm, 3 mm and 3.8 mm) was used to make a hole of 3.8 mm along the medullary cavity from the cut end of the tibia. The drilling resulted in a through hole at the proximal end and a blind-ended hole in the distal end. The length of the proximal-to-distal hole, including the bone scaffold, was the same as the interlock nail length. The bone scaffold was placed in the bone defect. The nail was then inserted into the medullary cavity through the bone scaffold using the targeting jig. Three side holes were drilled for the screws to fasten the interlocking nails with bone using the targeting jib side-hole alignment guide fixture. The fixture ensured that the screws were coaxially aligned with the central axis of the bone and the screw went through the pre-existing hole in the nail. A similar procedure was followed for the 3D-printed in vitro rabbit tibia model to check the efficacy of targeting jig.

### 2.6. Statistical Analysis

A one-factor analysis of variance (ANOVA) with subsampling, assuming unequal variances, was performed using the statistical tools of KaleidaGraph software 4.03 (Synergy Software, Reading, PA, USA) to determine if there was any significant effect of alginate application on the mechanical and biological functions of PCL. For all statistical tests, *p* < 0.05 was considered as the statistically significant comparison.

## 3. Results

### 3.1. Fabrication of Bone Scaffold, Scaffolds, Interlock Nails and Screws

**Figure 7:**
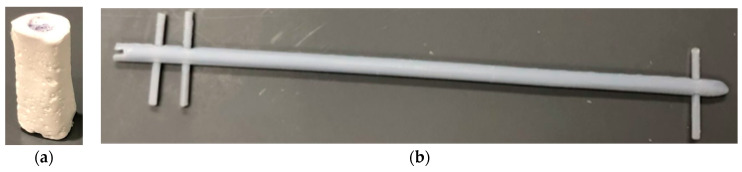
(**a**) Produced PCL bone scaffolds (**b**) intermodular screw and interlock nails using the phase separation casting method.

The casting method using the phase-separation technique was successful in fabricating microporous scaffolds made of PCL. The shape of these scaffolds required some minor post-processing due to defects in the silicone molds, but overall, this method produced viable results for producing bone scaffolds, interlocking nails and screws, as shown in Figure 7.

### 3.2. Surface Characterization

**Figure 8:**
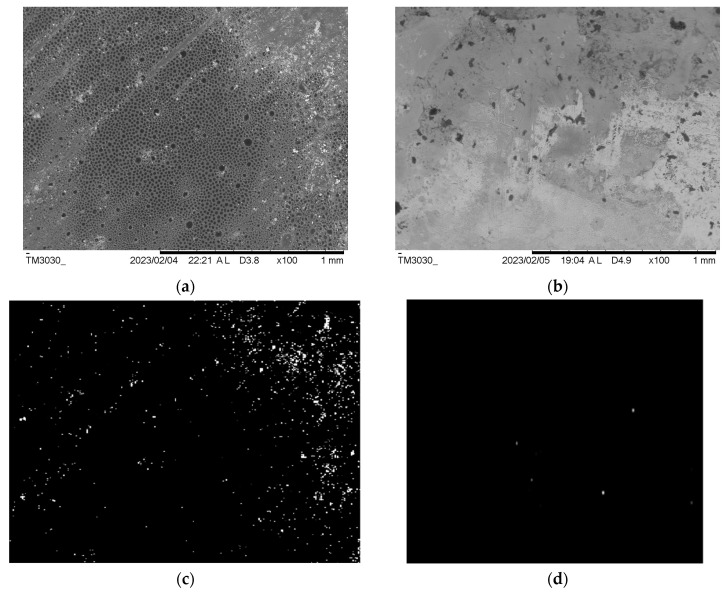
SEM images of the top surface of a (**a**) PCL and (**b**) PCL-Alg scaffold. MatLab binary images showing the white pixel as the hole and the black pixel as the solid area from the SEM images of (**c**) PCL and (**d**) PCL-Alg scaffold.

The SEM surface images and pore distribution analysis of the PCL and PCL-Alg scaffolds are shown in Figure 8. Fewer surface pores were observed on the PCL scaffold (Figure 8a) compared to PCL-Alg (Figure 8b) due to the integration of alginate into the PCL. We also observed higher porosity in the PCL scaffold compared to the PCL-Alg scaffold. The engrossed alginate with PCL can be seen in the PCL-Alg scaffold (Figure 8b). MATLAB image processing analysis of the PCL scaffold found that an average 11% surface area of the image contained pores. Due to infiltration of alginate into the pores in PCL-Alg, image analysis was unable to accurately calculate the total surface pore area from the SEM image of the PCL-Alg scaffold. Pore size parameters varied across locations. The measured diameter of the pores ranged from 0.05 µm to 18 µm.

### 3.3. Mechanical Tests

**Figure 9:**
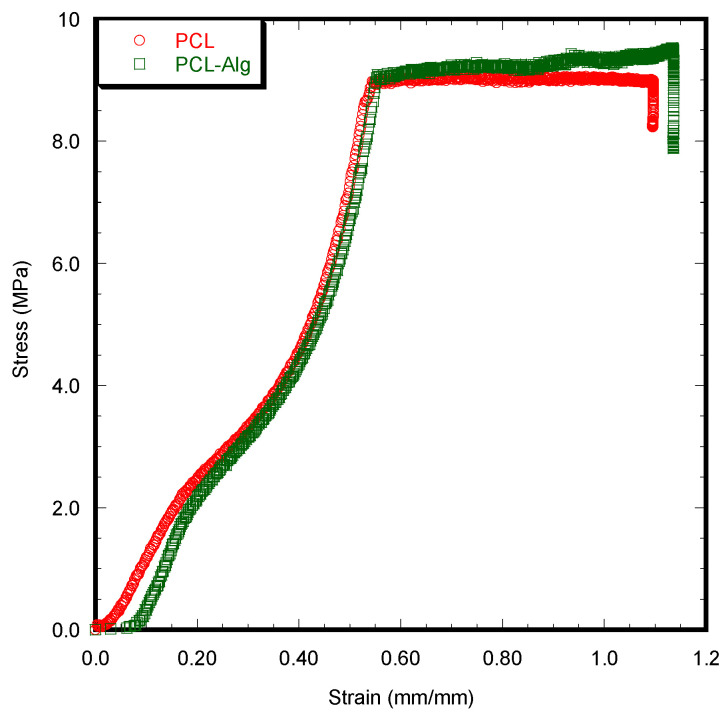
The stress vs. strain curve of PCL and PCL-Alg scaffolds during the static compression tests.

The PCL-Alg scaffold had higher compressive stress and modulus measurements compared to PCL (Figure 9). However, the difference in compressive strength and modulus between PCL and PCL-Alg scaffolds was not significant (Table 1). The results confirmed that both scaffolds had sufficient mechanical strength (compressive strength > 1.5 MPa) for the bone defect model.

### 3.4. Water Absorption and Degradation Test

The difference in the amount of water absorption and degradation between PCL and PCL-Alg after an hour was statistically significant (Table 2). Both sample groups degraded over the 7-day period. However, PCL degradation was very low after 7 days. The samples had a positive change in mass because they absorbed PBS. PCL-Alg samples degraded more compared to PCL due to the alginate hydrogel coating itself degrading, as shown in Table 2.

### 3.5. Cell Viablity Test

**Figure 10:**
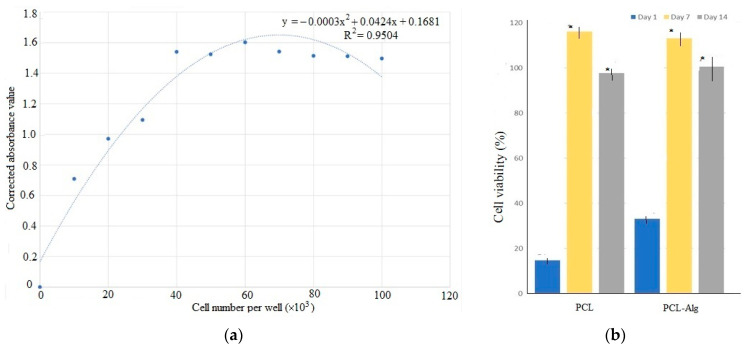
(**a**) The standard curve of absorption to cell number values found during the Alamar Blue cell viability assay. (**b**) The difference of cell viability in percentage between PCL and PCL-Alg measured on days 1, 7 and 14. In the figure, * indicates *p* < 0.05 compared to day 1 data.

A cell viability test was conducted using osteoblasts seeded on both types of samples. The standard curve in Figure 10a shows that the alamarBlue assay demonstrated a linear correlation between reduction and cell number within a range of 10,000 to 60,000 cells, reaching a maximum plateau around 60,000 cells; therefore, we plated less than half that amount (25,000 cells) per well to allow continued growth measurement. Figure 10b shows the initial results of the different types of samples on Days 1, 7 and 14. Cell viability results showed that the percentage of cell viability, which is correlated to the % reduction of alamar blue reagent, increased on Day 7 and decreased on Day 14 compared to Day 7. On Days 7 and 14, the percentage of cell viability was significantly higher compared to Day 1 (*p* < 0.05). Interestingly, the viability measurement decreased at 14 days; however, the decrease was not statistically significant (*p* > 0.5).

### 3.6. Target Jig Efficacy Tests

#### 3.6.1. Ex Vivo Demonstration

**Figure 11:**
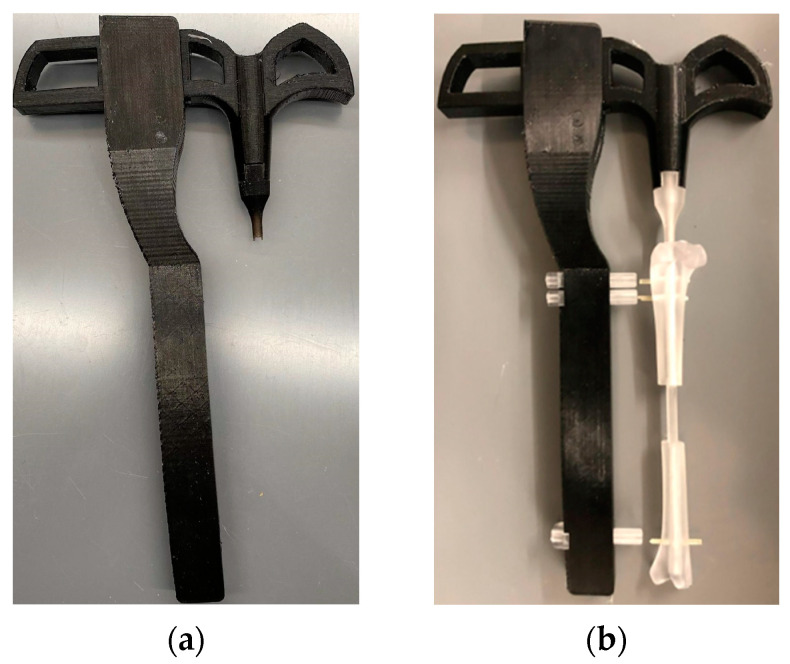
(**a**) The 3D printed targeting jig. (**b**) The efficacy tests of the manufactured delivery jigs using a 3D-printed rabbit tibia model showing that the jig can install an intermedullary nail and screw into a 20 mm-long rabbit tibia defect.

The targeting jig parts were fabricated and assembled, as shown in Figure 11. The handle and arm pieces were Formlabs FDM (extrusion) printed using biocompatible PLA material, while the fixator and channels were printed with Formlabs BioMed Amber Resins. Modeling the screw holes slightly larger than intended allowed for appropriate clearance of the joints, and the jig was successfully used to demonstrate the implantation of a resin-printed nail and screws into a resin-printed clear rabbit tibia model. The rabbit tibia model was 3D printed. A rabbit tibia model was cut 20 mm along the center of the tibia. Nails and screws were installed following the procedure given in Section 2.6 to test the accuracy of the fabricated parts before the cadaveric study.

#### 3.6.2. Cadaveric Tests

**Figure 12:**
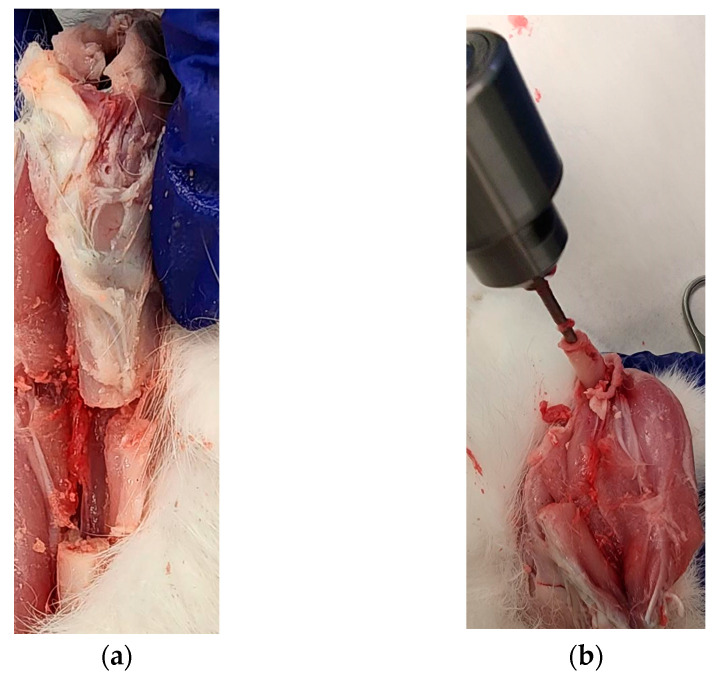

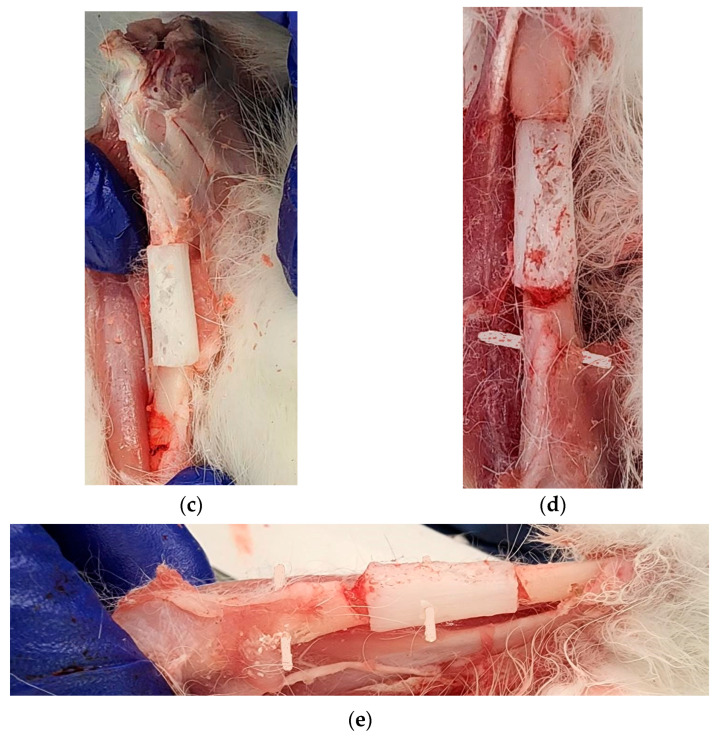
First leg: (**a**) A 20 mm long bone defect created by cutting the bone using an orthopedic oscillating bone saw; (**b**) Creating intramedullary holes for the nail; (**c**) An installed scaffold with the interlocking nails; and (**d**) The fastening of nails with tibia bone using a screw. Second leg: (**e**) Fastening of scaffold with a nail by a screw in an installed interlocking nail system in a long bone defect model.

Our cadaveric study successfully repaired a 20 mm long bone defect using the PCL bone scaffold, nails and screws in both legs of a rabbit following the procedure given in Section 2.5. Figure 12a–d shows the sets of steps followed for the installation of scaffold, nail and screws in one leg. Figure 12e shows the complete installation of the scaffold, nail and screws with an extra screw fastened through the scaffold with nail to show the drillability of the PCL scaffold and nails.

## 4. Discussion

According to the American Academy of Orthopedic Surgeons, there are approximately 6.3 million fractures each year in the United States, with more than 500,000 procedures of bone grafting costing roughly $2.5 billion [19]. However, limited availability, donor site pain, prolonged surgery time, and therefore an increased risk of infection have urged researchers to develop bone-grafting substitutes [20]. Critical size bone defects pose a significant clinical and socioeconomic problem, as they negatively impact patients’ quality of life due to consecutive reoperations and prolonged hospitalizations.

In this study, we successfully designed and prototyped a 3.2 mm diameter interlock nail and a 1.0 mm diameter fixation screw using an established polymer casting technique [14,15]. We manufactured a custom silicon mold to produce a porous PCL bone scaffold and coated PCL scaffolds with alginate to prepare the PCL-Alg scaffolds. Our water absorption, degradation and mechanical results found that both PCL scaffolds were porous and suitable for both water absorption and early weight-bearing. The porosity of the scaffold allowed the alginate hydrogel to infiltrate the scaffold. Our water absorption and degradation tests showed excellent water absorption and biodegradation profiles. The cell viability results showed that alginate had a positive influence on the cell viability of the PCL scaffold. We developed a targeting jig to assist with the alignment and accurate placement of the interlock nail and fixation. Our in vitro efficacy tests of targeting jig using a 3D-printed rabbit tibia and cadaver showed the potential of using the jig in a future in vivo study.

The alamar blue assay measures resazurin reduction when electrons are donated by NADH during the electron transport chain of aerobic cellular respiration [21]. It can correlate this activity with the number of viable cells present within a given time and cell number range [22,23]. The data presented here correlate well with increases seen when other cell types are cultured for extended periods of time. The measurement of higher cell viability in PCL-Alg compared to PCL only might be due to the fact that alginate facilitated the attachment of osteoblasts on the PCL surface, which led to better in vitro cell viability performance of PCL-Alg samples compared to PCL only. The sharp viability measurement increase in both PCL and PCL-Alg during 7 days of incubation demonstrates that the environment was optimized for osteoblast proliferation. The viability decrease at 14 days of incubation may not be correlated to cell number decrease but instead linked to reduced metabolic activity of osteoblasts because they are confluent or differentiating [24]. Our future study will address this question.

We measured the mechanical characteristics of PCL and PCL-Alg to evaluate their mechanical rigidity and whether they were comparable to native human bone. Our results revealed that the scaffold compressive strength of 9.51 ± 0.24 MPa closely matches that of the human cancellous bone (between 1.5 and 12 MPa) [25]. The mechanical stability of the small-scale interlock nail and screw fixation can further be improved if the silicone mold had fewer defects and if the constituent material had higher mechanical strength during its production. The targeting jig, while functional, may be improved by adjusting the channel size and a more stable design that provides more control over implant screw fixation.

The most significant challenge that we faced while developing the interlocking nail system was the size required to fit the small dimensions of the rabbit tibia. A higher mechanical stability was desired for the purpose of better weight bearing capability, and to that end, we attempted to produce a scaffold with a compressive strength (130–200 MPa) and compressive modulus (11.5–17 MPa) comparable to cortical bone [25]. The phase separation method was highly successful in producing a stronger and more stable scaffold. That, however, was not the case with the smaller and thinner pieces of an interlock nail and screws, and the resulting pieces had larger hollow sections than expected.

This study was limited to surface pore analysis. We will determine the porosity and interconnectivity of these pores in our future studies. After cell seeding, it will be essential to know the distribution of cells and whether the cells can infiltrate the scaffolds. However, this was beyond the scope of our in vitro cell studies. For our future in vivo study, we will examine cell infiltration using histological analysis.

The bone scaffold was designed for an eight-week-old rabbit. However, we used different-aged rabbits for our cadaver study. The mild mismatch does not hinder results due to the patient’s bone remodeling potential in vivo. In addition, our designed scaffold will be bioabsorbable; therefore, mild mismatch will not be an issue. Our future in vivo study goal is to use patient-specific bone scaffold modeling through a microcomputed tomography (µCT) scanned model of a 4.0 kg rabbit. This study did not measure the mechanical stability of an interlock system through mechanical tests. This study only assessed whether our designed systems could be implanted clinically on long bone defects. We will evaluate the interlock system’s mechanical stability through three-point bend and torsion tests after eight weeks of implantation for a long bone repair surgery.

Overall, the reconstruction of long-bone segmental defects is one of the most challenging tasks in orthopedic surgery. Current reconstructive methods are difficult, long and painful for both the surgeon and the patient. The proposed solution, using a bioabsorbable interlocking nail system and scaffold, offers many advantages, including additional stability, reduced recovery time, load-bearing during the recovery process, and no need for future surgical procedures owing to the bioabsorbable nature of the implants.

The project created a unique PCL porous scaffold with and without alginate hydrogel for a small animal model to aid cell growth in critical-size bone defects and an interlocking nail system to fix the scaffold in place and osseointegrate with native bone over time. The key innovation is designing an all-synthetic 3D-printed osteo scaffold and bioabsorbable interlock nails to tailor specific patient anatomy and bone size and location, permitting immediate postoperative motion and WB with gradual biodegradation, allowing creep substitution with regenerative bone tissue. The present work resulted in fundamental knowledge regarding the novel design and production of intramedullary nails and screws using biodegradable material for a rabbit tibia model. Additionally, this project created a targeting jig for delivering nails, screws and bone scaffolds for a rabbit tibia model, which has not yet been established. These produced prototypes are highly clinically translatable because all scaffold materials are USA FDA approved.

Among synthetic materials alternative to polymers, composites involving bioceramics based on calcium phosphates, hydroxyapatite and biodegradable polymer-chitosan have gained great popularity in recent years [26,27,28]. However, single materials do not usually provide the required mechanical, chemical and biological properties. The ideal candidates for composite scaffolds are polymers that gradually degrade while new tissue is being formed. Porous PCL filled with highly osteoconductive and osteoinductive hydrogel could be a potential solution. Hydrogel composed of nanoparticles, biomimetic apatite and natural polymers, such as collagen, are considered promising future biomaterials [29] for bone grafting. We believe that PCL-Alg, which has bioactive and bioresorbable properties, could be augmented further with collagen in order to accurately reproduce the bone microstructure and activate the mechanisms of bone tissue regeneration and thus to meet the requirements of regenerative medicine [30,31].

In summary, our findings (morphology, mechanical, porosity, degradation) showed that our design scaffold is structurally valid. However, an opportunity to optimize alginate or use alternative engineered gels (e.g., collagen) in the bone scaffold is possible to improve osteo- and chondro-conductivity and biocompatibility in vitro and in vivo. This will require reconstructing the prototype to ensure proper seeding and stability. In future studies, we aim to optimize this scaffold to have superior biomechanical properties, allowing immediate postoperative weight bearing in osteochondral lesion repairs, as well as superior histological and structural bone tissue repair. This may involve integration of formerly mentioned hydroxyapatite or a similar adjuvant to firstly assess hydroxyapatite biomodulation in PCL and which, to our knowledge, has not been investigated. Successful incorporation would further enhance cell viability and proliferation.

## 5. Conclusions

This study investigated a tibial reconstructive scaffold and interlocking nail system for a rabbit model with a critical-sized long-bone segmental defect. We concluded that (1) our created PCL and PCL-Alg have physical, mechanical and biocompatibility characteristics to be qualified as a tissue-engineered scaffold, (2) our PCL interlocking nail and screw have adequate weight bearing and can be press-fitted into a rabbit tibia medullary canal and proximal screw channels, and (3) our targeting jig can secure a bone scaffold and nail system in a rabbit tibia model. The first conclusion was based on water absorption, degradation, compression and cell viability tests on the PCL scaffold with and without alginate hydrogel. Our second and third conclusions were based on cadaver tests for accurate placement of the bone scaffold and fixation of the interlock nail system, tested using our developed targeting jig. The prototype developed in this study can be used for future in vivo reconstructive surgery on a rabbit tibia. The study models could provide an effective method to treat clinically large segmental bone defects.

## Figures and Tables

**Table 1:** Difference between the mechanical properties of the PCL scaffolds during compression tests. All samples had statistically insignificant diameter differences (average = 7.9 mm) and were loaded up to the full height of the sample to calculate the mechanical properties. Data presented *n* = 3 for both samples. Data are presented as a mean ± standard error. No statistically significant difference was observed between the sample groups (*p* > 0.05).

Experimental Parameters	PCL	PCL-Alg
Compressive Strength (MPa)	9.51 ± 0.24	10.34 ± 0.95
Compressive Modulus (MPa)	17.34 ± 1.39	23.41 ± 4.28

**Table 2:** Water absorption and degradation differences between PCL and PCL-Alg scaffolds. Data presented *n* = 3 for both samples. Data are presented as a mean ± standard error. In the table, * indicates *p* < 0.05 compared to PCL and the negative sign represents the decrease of weight compared to initial weight.

Sample Type	Water Absorption (%)	Degradation after 7 Days
PCL	14.54 ± 5.51%	−0.08 ± 0.10%
PCL-Alg	35.53 ± 0.92% *	−1.18 ± 0.26% *

## Data Availability

Upon request via email of the corresponding author.
